# Author Correction: Prior Experience Alters the Appearance of Blurry Object Borders

**DOI:** 10.1038/s41598-020-75461-3

**Published:** 2020-11-20

**Authors:** Diana C. Perez, Sarah M. Cook, Mary A. Peterson

**Affiliations:** 1 Psychology Department, University of Arizona, Tucson, AZ USA; 2 Cognitive Science Program, University of Arizona, Tucson, AZ United States

Correction to: *Scientific Reports* 10.1038/s41598-020-62728-y, published online 2 April 2020

In the original version of this Article, Sarah M. Cook and Mary A. Peterson were incorrectly affiliated with ‘Department of Psychology, Northwestern University, Evanston, IL, United States’. The correct affiliation is listed below.

‘Psychology Department, University of Arizona, Tucson, AZ, USA’.

This error has now been corrected in the PDF and HTML versions of the Article.

